# Study of *Lavandula dentata*, *Salvia rosmarinus*, and *Cymbopogon citratus* essential oils profile and antifungal activity of their mixture against the gray mold *Botrytis cinerea*

**DOI:** 10.3389/fpls.2025.1694585

**Published:** 2025-11-06

**Authors:** Salahddine Chafiki, Abdallah Oukarroum, Redouan Qessaoui, Soumaya El Assri, Mohamed Alouani, Hasnaa Lahchimi, Hicham El Arroussi, Rachid Bouharroud

**Affiliations:** 1 Regional Center of Agricultural Research of Agadir, National Institute of Agricultural Research (INRA), Rabat, Morocco; 2 AgroBioSciences Department (AgBS), Mohammed VI Polytechnic University (UM6P), Ben Guerir, Morocco; 3 Research Team in Science and Technology, Higher School of Technology, Ibn Zohr University, Laayoune, Morocco; 4 Laboratory of Biotechnology and Valorization of Natural Resources, Faculty of Science, Ibn Zohr University, Agadir, Morocco; 5 Faculty of Applied Science, Ait Melloul, Ibn Zohr University, Agadir, Morocco; 6 Algal Biotechnology Center, MAScIR, Mohammed VI Polytechnic University (UM6P), Ben Guerir, Morocco

**Keywords:** antifungal activity, B. cinerea, essential oil, mixture, tomato

## Abstract

In this study, we investigated the chemical profile essential oils (EOs) extracted from *Cymbopogon citratus*, *Salvia rosmarinus*, and *Lavandula dentata*, as well as their antifungal activity against *Botrytis cinerea in vitro* and *in vivo*. GC–MS analysis showed that the EOs major components of *C. citratus* EOs were, Geranial (42.91%), Neral (34.11%), and β-Pinene (9.32%). While the *S. rosmarinus* major EOs components were Camphor (17.60%), α-Pinene (14.39%), and 1,8-cineol (14.13%). Contrariwise *L. dentata* EOs, Camphor (33.95%), 1,8-cineol (32.35%), and β-Pinene (5.23%) were the predominant compounds. Regarding the *in vitro* antifungal activity, the EOs of three plants inhibited the mycelial growth of *B. cinerea* in a dose-dependent manner. Moreover, at the concentration of 0.32 µL/mL air, all EOs demonstrated the inhibition of the mycelia growth of *B. cinerea*. In addition, the combination of EOs increased the antifungal activity of *B. cinerea* compared to their individual application. According to simplex-centroid design analysis, the most efficient antifungal of the mixture of EOs extracted from *L. dentata*, *S. rosmarinus*, and *C. citratus* was noted EOs at a ratio (1:1:1). This mixture inhibited the mycelial growth at 1.6 µL/mL, with IC_50_ and IC_90_ value of 0.46 µL/mL and 0.81 µL/mL, respectively. In addition, *in vivo* tests showed that this EOs mixture significantly reduced the decay of cherry tomatoes caused by *B. cinerea* with an average of 88.37%. Also, the disease severity value recorded for the plant treated with the EOs mixture was 19.29% compared to the control with an average of 88.57%. This study demonstrates that the mixture of *L. dentata*, *S. rosmarinus*, and *C. citratus* EOs is a promising natural antifungal agent for managing *B. cinerea* infections.

## 1 Introduction

EOs are complex mixtures of volatile, organic compounds generated by plants, and they are accountable for a plant’s distinctive flavor and fragrance (Tisserand and Young, 2014). EOs’ main compounds are categorized under two structural components: terpenoids and phenylpropane (Ni et al., 2021). For centuries, EOs were appreciated for their biological, antimicrobial, and other beneficial effects in medicine (Ni et al., 2021), and they’re currently applied in additional fields from cosmetics and food industries to agriculture (Moghaddam and Mehdizadeh, 2017). For example, EOs compounds can successfully inhibit the growth of microorganisms (Hammer et al., 1999; Koroch et al., 2007), which make them potential candidates for controlling plant pathogens (Isman, 2000; Berrada et al., 2012; Medina-Romero et al., 2021; Tagnaout et al., 2023). EOs and their components are also valued for their availability and their low cost (Mutlu-Ingok et al., 2020).

In recent years, studies have indicated efficiency against phytopathogenic fungi such as *B. cinerea*, the second most important phytopathogenic fungus worldwide (Dean et al., 2012) and the causal agent of grey mold, a standout post-harvest disease associated with tomato production (Lahmyed et al., 2021; Alkilayh et al., 2024). The losses caused by this pathogen can amount to 1 billion euros a year (Dean et al., 2012) and it’s a serious threat to many croups production in Morocco (Berrada et al., 2012; Qessaoui et al., 2024).

Based on the literature, EOs from various plant species indicated efficiency against this pathogen (Bouchra et al., 2003; Soylu et al., 2010; de Oliveira Filho et al., 2021; El Abdali et al., 2022; He et al., 2023; de Albuquerque Sousa et al., 2024; Ramos et al., 2024; Bouqellah et al., 2025). EOs lavender, lemongrass, mint and others were tested in vapor phase against *B. cinerea* and concluded ability to suppress the fungus (Tančinová et al., 2022). *Syzygium aromaticum* and *Brassica nigra* and their combination were found to exhibit inhibitory effect against the pathogen *in vitro* and *in vivo* (Aguilar-González et al., 2015). The monoterpenes carvacrol and thymol induced conidial death of *B. cinerea* (Pedroso et al., 2024), and EOs derived from *Pelargonium roseum* inhibited the mycelium growth and spore germination of *B. cinerea* in cherry tomato (Fuyan et al., 2025). EOs can also be applied in synergy with other EOs to increase their biological effect and reduce EOs doses (Targino de Souza Pedrosa et al., 2021; Milagres de Almeida et al., 2023).

The present study aims to investigate EOs extracted from the aerial parts of three plant species grown in south-eastern Morocco: *L. dentata* and *S. rosmarinus* from Lamiaceae family and *C. citratus* belonging to the Poaceae; and to test their synergistic effect *in vitro* and *in vivo* against *B. cinerea*. The findings intend to further knowledge on the synergism between different EOs and their multiple active compounds.

## 2 Materials and methods

### 2.1 Preparation of the pathogen

*Botrytis cinerea* (PX434395) was isolated from infested leaves of tomato in the Chouka Ait Baha region, Morocco. Isolate characterization was carried out at the Plant Protection Laboratory of INRA Agadir, Morocco. Identification of *B. cinerea* was conducted based on morphological, microscopic, and molecular characteristics (Gull and Trinci, 1971; Blancard, 2012). The fungus was maintained in potato dextrose agar (PDA) and stored at 4°C until use.

### 2.2 Extraction and gas chromatography–mass spectrometry analysis of EOs

Three plants (*C. citratus, L. dentata, and S. rosmarinus*) were collected at the flowering stage from the experimental farm of the National Institute for Agricultural Research (INRA), Agadir, Morocco (30°02′42.2”N 9°33′13.4”W) in May 2023. The plants were identified and confirmed by botanists from INRA in Agadir and Ibn Zohr University, Morocco. EOs were extracted from air-dried plant materials by hydro-distillation for 3 h using a Clevenger-type apparatus. Following extraction, the EOs were dried using anhydrous sodium sulfate and stored in sealed vials at temperature (4°C) until analysis (Soltanbeigi and Maral, 2025). The yields were calculated using the following formula:


$$\text{Yield }(\%)=\frac{\text{Amount of EO recovered }(\text{g})}{\text{Amount of plant material distilled }(\text{g})}\;\times 100$$

The GC–MS analysis was performed using GCMS-TQ8040 SHIMADZU, JAPAN. Rtx^®^-5MS fused-bond column (30 m length, 0.25 mm internal diameter, and 0.25 µm film thickness, Restek, PA, USA) was installed. The starting temperature was 50°C (2min) to 300°C with a ramp of 5°C/min and an isotherm at 300°C for 3 min. The injector temperature was set at 250°C and helium with a flow rate of 1.5 mL/min was used as carrier gas. The conditions for the mass spectra were as follows: Ion source temp 200°C, Interface temp: 280°C, Mass range: 50–500 m/z, electron ionization (EI) at 70 eV. The samples were filtered using a syringe filter (0.45 µm). One μL of diluted samples (1:10 hexane, v/v) was injected in split mode. The analysis of compounds was identified with the database NIST 2017, and the retention indices were determined via n-alkanes standards (Mahmoud et al., 2023).

### 2.3 Optimization of the antifungal activity of the EOs using the methodology of mixture design

#### 2.3.1 Determination of the *in vitro* antifungal activity of EOs

Antifungal activity of the three EOs and their mixtures (ratio 1:1:1) on mycelial growth of *B. cinerea* was determined by the volatile activity (VA) assay as described by Basaid et al. (2021) with some modifications. Sterilized Petri dishes (inner diameter 90 mm) were filled with 20 mL of PDA, and one mycelium plug (5-mm diameter) was placed on the PDA in the center of the Petri dishes. The EOs were pipetted onto sterile Whatman 1 filter paper discs (25-mm diameter) at the concentrations of (C1 = 0.02, C2 = 0.04, C3 = 0.08, C4 = 0.16, and C5 = 0.32 µL/mL air of Petri dish). The treated discs were attached to Petri dish lids, and the Petri dishes were sealed with parafilm and incubated at 24 ± 1°C for 7 days. The mycelial growth inhibition (MGI) rate was calculated using the formula described by Pandey et al. (1982):


$$MGI\;(\%)=\frac{\text{Dc}-\text{Dt}}{\text{Dc}}\;100$$

where Dc and Dt represent mycelial growth diameter in control and treated Petri dishes, respectively. The treatments were replicated three times.

#### 2.3.2 Experimental design

A simplex-centroid design was adopted to optimize the mixture of the EOs of *L. dentata*, *S. rosmarinus*, and *C. citratus* as outlined by Benkhaira et al. (2023). The components of the EOs system are detailed in **Table 1**. The proportion of each EO in the mixture can range in value from 0 to 1, with the total sum of the three components equaling 1. The antifungal activity of the EOs was evaluated using the inhibition rate and the half maximal inhibitory concentration (IC_50_).

**Table 1:** Design of the three components of the EOs mixture.

Mixture	*L. dentata*	*S. rosmarinus*	*C. citratus*
1	1	0	0
2	0	1	0
3	0	0	1
4	0.5	0.5	0
5	0.5	0	0.5
6	0	0.5	0.5
7	0.33333	0.33333	0.33333

#### 2.3.3 Experimental matrix and mathematical model

**Figure 1:**
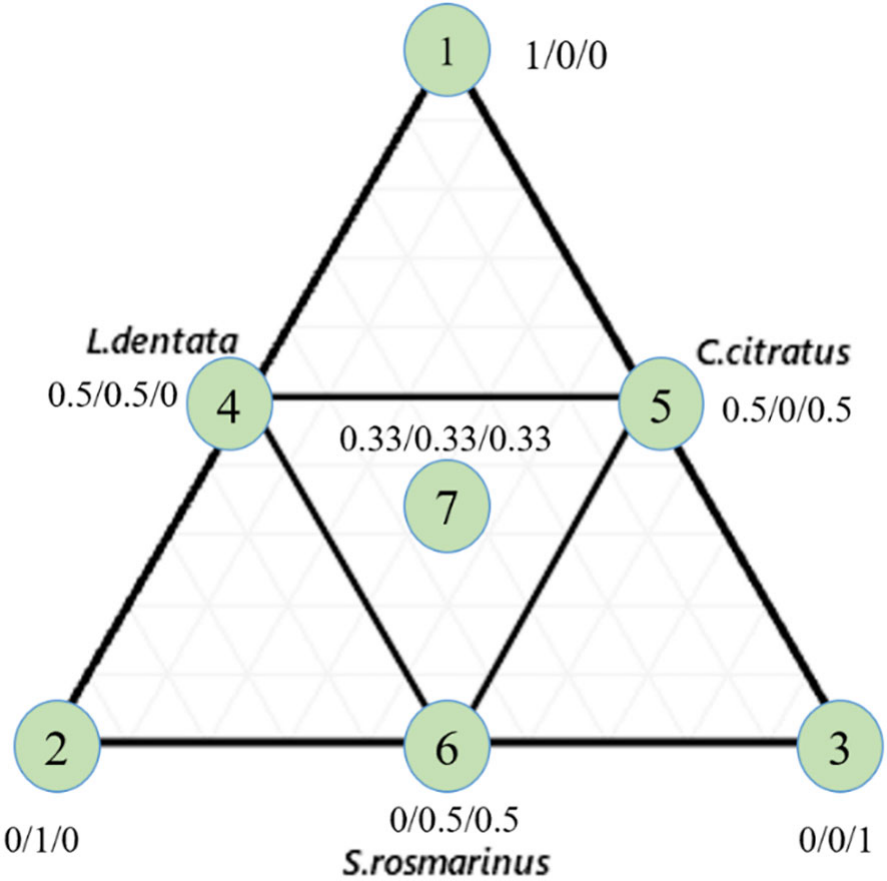
Equilateral triangle of the arrangement of mixtures using the simplex centroid design method. 1: L. dentata EO; 2: S. rosmarinus EO; 3: C. citratus EO.

Seven compositions are represented by an equilateral triangle (**Figure 1**). The triangle vertices (X1, X2, X3) correspond to the pure oils (100%), the binary mixtures (50%/50%) are conforming to midpoints of three sides of the triangle (X4, X5, X6), and equi-proportional mixture of the three oils (33.33%/33.33%/33.33%) is fixed in the center of the triangle (X7). The special cubic model was applied to assert responses based on the independent variables as represented by the following equation:


$$\begin{array}{c} Y=a_{1}X_{1}+a_{2}X_{2}+\;a_{3}X_{3}\;+\;a_{12}X_{1}X_{2}\;+a_{13}X_{1}X_{3}\;+\;\\ a_{23}X_{2}X_{3}\;+a_{123}X_{1}X_{2}X_{3}\;+\;\varepsilon \end{array}$$

where Y is the experimental response (IC_50_). The α1, α2, and α3 are linear regression coefficients, α12, α23 and α23 are binary regression coefficients, and α123 is the ternary regression coefficient, while ε is the regression error term (Benkhaira et al., 2023).

#### 2.3.4 Mixture design analysis

The statistical significance of the mathematical model was assessed at a 95% confidence level using the F-ratio mean square regression/mean square residual, which compares the mean square regression to the mean square residual; higher F-values indicate greater variability in the results (EL Hachlafi et al., 2023). Additionally, the ratio of the mean square lack of fit to the mean square pure error was analyzed to evaluate the model’s adequacy, with higher values suggesting potential inadequacies. The coefficient of determination (R²) was calculated to assess model quality. The significance of estimated factors was evaluated using the Student’s t-test, while ANOVA’s F-test confirmed the overall model significance. Analyses were conducted using Design Expert software version 23.1 and Minitab^®^ 19.2020.1. For optimization, contour plots and 3D surface plots illustrated trade-off areas among the studied components. The desirability tool was used to identify the optimal values, balancing factors for the best outcome. This tool adjusts the mathematical model within a range of 0 to 1, where 0 indicates an undesirable response and 1 signifies a highly desirable response.

### 2.4 Antifungal assay with the selected mixture

#### 2.4.1 *In vitro* direct contact assay

The EOs mixture that showed a significant effect after the simplex-centroid design analysis was selected. Antifungal activity was evaluated by incorporating the EOs mixture into the culture medium. The EO was added to sterilized PDA to obtain the following range of concentrations: 0.2, 0.4, 0.8, 1.6, 3.2 μL/mL. The plates were inoculated with the fungus, using a 5 mm diameter agar disk. The agar plates were then incubated for 7 days at 24 ± 1°C. The antifungal activity was expressed by the percentage of mycelial growth inhibition and calculated according to the formula described in section 2.3.1. The treatments were replicated three times.

#### 2.4.2 Minimum inhibitory concentration and minimum fungicidal concentration

MIC was determined by the broth microdilution method. One hundred μL of Sabouraud dextrose broth (SDB) was added to the 96-well plates, and a serial twofold dilution of the EOs mixture was prepared to obtain concentrations from 0.2 to 3.2 μL/mL. Then, 100 μL of spore suspension (10^5^ spores/mL) was added to each well. The control was set up using SDB with spores. Plates were incubated at 24 ± 1°C for 24 h. The MIC is the lowest concentration that prevents *B. cinerea* growth. The MFC was determined by transferring 100 μL of the mixture which showed the lowest concentration without mycelial growth to PDA plates. The plates were incubated at 24 ± 1°C for 72 h. The MFC is the concentration at which *B. cinerea* showed no growth (Wiegand et al., 2008).

#### 2.4.3 Spore germination assay

*Botrytis cinerea* spore suspension (10^5^ spores/mL) was incorporated into a solution of Sabouraud dextrose broth and EOs mixture at different concentrations (0.2, 0.4, 0.8, 1.6, 3.2 μL/mL) at equal ratio (1:1, v/v). In the control treatment, the same volume of spores inoculated was used without the EOs. The tubes were incubated at 24 ± 1 °C for 24 h. Then the spore germination was visualized with a light microscope at 400 × magnification, and a minimum of 100 spores from each replicate were analyzed. Spores were considered germinated when the size of the germ tube surpassed the largest diameter of the spore. The inhibition of spore germination (IG) was calculated using the following formula:


$$\text{IG }(\%)=\frac{\left(\text{Gc}-\text{Gt}\right)}{\text{Gc}}\times \;100$$

Gc and Gt are, respectively, the averages of spores germinated in controls and treatment tubes. For each concentration, five replicates were assessed (Soylu et al., 2010).

#### 2.4.4 *In vivo* assay on cherry tomato fruits

The effect of the EOs mixture on *B. cinerea in vivo* was assessed using a previously reported method by Yang et al. (2023) with some modifications. The fruits were disinfected with 0.01% sodium hypochlorite for 2 min and air-dried. Each fruit was punctured at two points (3 mm deep and 3 mm wide), and 10 μL of spore suspension (10^5^ spores/mL) was pipetted into the wound. The fruits were air-dried for 1 h, then 10 μL of the mixture at 0.81 μL/mL (IC_90_) of EOs emulsified in Tween 80 (0.1%) solutions was added to each wound. Control samples were pipetted with 10 μL of sterile Tween 80 solution. The samples were stored in plastic containers at 24 ± 1°C for 7 days. Each treatment contained three replicates with 10 fruits per replicate. The proportion of rot lesions was calculated using the following formula:

$\text{Fruit decay area }({\text{mm}}^{2})=\text{π}\times \text{a}\times \text{b}$


$$\text{Inhibition rate }(\%)=\frac{\text{Dc}-\text{Dt}}{\text{Dc}}\;100$$

Where, Dc is mycelial growth diameter in control, and Dt is mycelial growth diameter in treated fruits, while a and b represent the vertical and horizontal lesion ray (mm).

#### 2.4.5 *In vivo* assay on tomato plants

IC_90_ of the EOs mixture was selected for the *in vivo* trial. The EOs were prepared by dissolving the requisite amounts in Tween 80 (0.1%) solution. Tomato plants were arranged in a split-plot design with three replicates of 6 plants per treatment. The plants were inoculated with a suspension of 10^5^ spore/mL of *B. cinerea*. After 24 h, tomato plants were sprayed with the emulsion of EOs (10 mL for each plant) uniformly with a manually operated sprayer, while the control plants were sprayed with Tween 80 (0.1%) solution. Control and EOs-treated plots were assessed 3 weeks after treatment. The disease severity index of grey mold on the tomato leaves was rated on a scale of 0–5 (0 = no disease symptom, 1 = 0.1–20%, 2 = 20.1–40%, 3 = 40.1–60%, and 4 = 60.1–80%, 5 = 80.1-100%) as the percentage of diseased leaf area (Lee et al., 2006).

### 2.5 Statistical analysis

The data were analyzed using RStudio (Posit Software, PBC. 2024.04.2). The comparison of means was performed using the Tukey, Kruskal wallis and Mann-Whitney U tests (p<0.05). The IC_50_ and IC_90_ of EOs and their mixtures were calculated using GraphPad Prism 10 version 10.3.1 (509) 2024.

## 3 Results

### 3.1 Chemical composition of EOs

The EOs yields obtained by hydrodistillation for *S. rosmarinus*, *L. dentata*, and *C. citratus* were 0.97 ± 0.06%, 0.91 ± 0.04% and 1.02 ± 0.02%, respectively. The chemical profiles of the EOs of the three medicinal plant species are summarized in **Table 2**. A total of 102 volatile compounds were identified from the GC/MS analyses. Thirty-one volatile compounds were identified in the oil of *C. citratus* leaves, in which oxygen-containing monoterpene represented the most prevailing class, constituting 61.29% of the oil, followed by monoterpene hydrocarbons (12.90%). Geranial (42.91%), neral (34.11%), and β-pinene (9.32%) were the most abundant compounds. Furthermore, geraniol, isogeranial, and linalool were present at considerable quantities, representing 2.42, 2.13 and 1.33%, respectively.

**Table 2:** Chemical compounds of the EOs of C. citratus, L. dentata, and S. rosmarinus.

Name compounds	Retention index		Relative abundance (%)		
	Calculated	Reported	*C. citratus*	*L. dentata*	*S. rosmarinus*
Cyclene	924	919	–	–	0.16
α-Pinene	935	936	–	1.43	14.39
Camphene	950	948	–	0.50	3.43
Dehydrosabinene	956	956	–	–	0.84
1,3,5-Cycloheptatriene, 3,7,7-trimethyl-	972	970	–	–	0.05
Sabinen	975	975	–	0.59	–
β-Pinene	979	977	9.32	5.23	0.75
1-Octen-3-ol	981	986	–	–	0.28
Sulcatone	989	987	0.73	–	–
3-Octanone	989	993	–	–	0.10
β-Myrcene	993	993	–	–	0.98
3-Carene	1012	1014	–	–	1.00
4-Carene	1019	1022	–	–	0.29
β-Cymene	1027	1026	–	0.27	2.43
Limonene	1031	1031	0.15	2.02	0.00
1,8-cineol	1034	1033	–	32.35	14.13
Benzeneacetaldehyde	1047	1049	–	–	0.04
Beta-Ocimene	1050	1050	0.07	–	–
α-Toluenol	1059	1057	–	0.11	–
γ-terpinene	1061	1063	–	0.03	0.44
Sabinene hydrate	1070	1070	–	0.09	–
Linalool oxide	1075	1073	–	0.19	0.13
Rosefuran	1099	1093	0.14	–	–
L-Fenchone	1092	1096	–	1.85	–
Linalool	1102	1104	1.33	2.92	4.27
Perillen	1103	1102	0.41	–	–
Filifolone	1107	1107	–	–	0.65
Fenchol	1117	1114	–	0.49	0.12
2-Cyclohexen-1-ol, 1-methyl-4-(1-methylethyl)-, trans-	1125	1125	–	–	0.06
Chrysanthenone	1130	1128	–	–	0.85
Limona ketone	1135	1131	–	0.09	–
Pinocarveol	1144	1146	–	1.32	0.25
Cis-Verbenol	1146	1148	–	0.04	0.14
6-Octenal, 7-methyl-3-methylene-	1148	1146	0.50	–	–
Camphor	1150	1152	–	33.95	17.60
Cis-Limonene hydrate	1153	1153	–	0.05	–
Trans-Chrysanthemal	1154	1153	0.20	–	–
Citronellal	1156	1153	0.08	–	–
Ocimenol	1160	1155	0.08	–	–
Isoneral	1167	1175	0.87	–	–
Pinocarvone	1168	1167	–	0.59	0.32
Borneol	1171	1172	–	2.79	11.87
Trans-Pinocamphone	1179	1173	–	–	1.87
Rosefuran epoxide	1179	1177	0.08	–	–
L-terpinen-4-ol	1182	1185	–	0.42	1.25
Isogeranial	1185	1184	2.13	–	–
Cymen-8-ol	1189	1187	–	–	0.26
α-terpineol	1195	1191	–	1.63	2.78
Myrtenol	1201	1194	–	1.50	0.51
Cis-Carveol	1211	1217	0.10	–	–
Cis-Piperitol	1212	1196	–	–	0.05
Levoverbenone	1215	1212	–	0.09	10.42
Carveol	1223	1225	–	0.15	0.14
Citronellol	1232	1228	0.49	–	0.15
2,3-Epoxy-geranial	1237	1236	0.17	–	–
Neral	1246	1249	34.11	–	–
Carvone	1250	1249	–	0.12	–
Geraniol	1258	1263	2.42	–	0.32
Cis-Myrtanol	1266	1266	–	–	0.07
Geranial	1276	1278	42.91	–	–
Bornyl acetate	1291	1295	–	–	2.92
Carvacrol	1296	1297	–	–	0.05
2-Undecanone	1296	1298	0.82	–	–
Cuminol	1296	1298	–	0.07	–
Neryl formate	1305	1307	0.20	–	–
Neric acid	1326	1347	0.25	–	–
Hexyl tiglate	1334	1331	–	0.45	–
Piperitenone	1348	1343	–	–	0.69
Geranic acid	1358	1354	0.67	–	–
m-Eugenol	1363	1361	–	–	0.13
Trans-Sobrerol	1364	1382	0.27	–	–
α-Copaene	1383	1376	–	–	0.14
Geranyl acetate	1386	1383	0.20	–	–
Hexyl caproate	1388	1387	–	0.07	–
Methyleugenol	1409	1405	–	–	0.15
Caryophyllene	1428	1422	–	0.43	1.05
Cis-α-Bergamotene	1441	1414	0.08	–	–
Trans-α-Bergamotene	1442	1440	–	0.81	–
Humulene	1449	1453	–	0.26	–
Trans-Muurola-3,5-diene	1454	1450	–	0.16	–
Cis-Geranylacetone	1457	1457	–	–	0.17
(E)-β-Famesene	1460	1460	–	0.15	–
Cis-Muurola-4(15),5-diene	1472	1470	–	0.23	–
Dehydrosesquicineole	1475	1473	–	0.10	–
γ-Muurolene	1484	1480	–	–	0.13
(+)-Valencene	1495	1499	–	–	0.05
2-Tridecanone	1498	1496	0.73	–	–
α-Muurolene	1508	1500	–	–	0.05
β-Bisabolene	1514	1518	–	1.13	0.05
δ-Cadinene	1532	1523	–	–	0.31
Cis-Calamene	1532	1529	–	1.19	–
(E)-α-Bisabolene	1549	1547	–	2.81	–
α-Calacorene	1553	1546	–	–	0.04
Caryophyllene oxide	1594	1589	0.18	0.85	0.38
Humulene epoxide 2	1621	1614	–	–	0.11
Di-epi-1,10-cubenol	1626	1617	0.16	–	–
Selin-6-en-4.alpha.-ol	1630	1636	0.21	–	–
Epicubenol	1639	1620	–	–	0.08
τ-Muurolol	1665	1644	–	–	0.06
Bisabolol oxide B	1665	1666	–	0.22	–
Cadalene	1679	1674	–	0.09	–
2-Pentadecanone	1702	1703	0.08	–	–
Monoterpene hydrocarbons			12.90	15.91	21.43
Sesquiterpene hydrocarbons			3.23	20.45	16.07
Oxygen-containing sesquiterpene			6.45	11.36	7.14
Fatty acid esters			6.45	4.55	0.00
Oxygen-containing monoterpene			61.29	47.73	53.57
Others			9.68	0.00	1.79

Regarding *L. dentata* EOs, 44 volatile compounds were identified in which oxygen-containing monoterpene and sesquiterpene hydrocarbons constituted the major volatile compounds, representing 47.73 and 20.45% of the oil content, respectively. Camphor (33.95%), 1,8-cineol (32.35%), and β-pinene (5.23%) were the most abundant. Moreover, phytochemicals included linalool (2.92%), (E)-α-bisabolene (2.81%), α-terpineol (1.63%), borneol (2.79%), limonene (2.02%), α-pinene (1.43%).

Fifty-six compounds of *S. rosmarinus* EO were identified. Oxygen-containing monoterpene predominated with a value of 53.51%, followed by monoterpene hydrocarbons (21.43%) and sesquiterpene hydrocarbons (16.07%). The major components were camphor (17.60%), α-pinene (14.39%), and 1,8-cineol (14.13%), followed by borneol (11.87%), levoverbenone (10.42%), and linalool (4.27%). In addition, amphene (3.43%), bornyl acetate (2.92%), α-terpineol (2.78%), and β-cymene (2.43%).

### 3.2 Optimization of the antifungal activity of the EOs using the methodology of mixture design

#### 3.2.1 Determination of the *in vitro* antifungal activity of EOs

The results of *in vitro* antifungal activities of the EOs and their mixtures on *B. cinerea* are shown in **Table 3**. The application of all mixtures inhibited the mycelial growth of *B. cinerea* significantly in a dose-dependent manner (P< 0.001). Furthermore, all EOs mixtures showed higher inhibition compared to pure EOs. The concentration C5 in all EOs exhibited total inhibition of mycelial growth. The activity of EOs in the concentrations C4 and C3 was as follows: For *S. rosmarinus*, 80.69% and 55.30%, respectively; 72.05 and 46.79, respectively for *C. citratus*; and it reached 63.19% and 56.35%, respectively in the case of *L. dentata.* Concentration C1 demonstrates the lowest effect on fungal growth with an average of 8.74%, 18.20%, and 19.33% for *S. rosmarinus*, *C. citratus*, and *L. dentata*, respectively.

**Table 3:** Matrix of simplex centroid design and results for *in vitro* antifungal activity of EOs against mycelial growth of B. cinerea (Concentrations: C1-C5).

Mixture	*L. dentata*	*S. rosmarinus*	*C. citratus*	C1	C2	C3	C4	C5	P-value
1	1	0	0	19.33 ± 4.04^d^	42.72 ± 12.02^c^	56.35 ± 4.39^b^	63.19 ± 10.9^b^	100 ± 0.00^a^	<0.001
2	0	1	0	8.74 ± 1.16^e^	37.50 ± 3.13^d^	55.30 ± 13.58^c^	80.69 ± 13.0^b^	100 ± 0.00^a^	<0.001
3	0	0	1	18.16 ± 1.94^e^	36.33 ± 3.88^d^	46.79 ± 1.63^c^	72.05 ± 7.43^b^	100 ± 0.00^a^	<0.001
4	0.5	0.5	0	21.77 ± 1.97^c^	69.87 ± 1.59^b^	100 ± 0.00^a^	100 ± 0.00^a^	100 ± 0.00^a^	< 0.001
5	0.5	0	0.5	16.90 ± 8.76^d^	47.02 ± 1.37^c^	89.50 ± 0.57^b^	100 ± 0.00^a^	100 ± 0.00^a^	< 0.001
6	0	0.5	0.5	22.83 ± 1.63^c^	36.98 ± 2.32^b^	100 ± 0.00^a^	100 ± 0.00^a^	100 ± 0.00^a^	< 0.001
7	0.33	0.33	0.33	64.59 ± 12.79^c^	80.19 ± 8.08^b^	100 ± 0.00^a^	100 ± 0.00^a^	100 ± 0.00^a^	< 0.001

*By line, values with the same letters are not significantly different according to the Tukey test at 5%.

To examine the effect of synergism on the antifungal activity of the EOs, different ratios of mixtures were tested. The results indicate that the inhibition rate increased from 21.77% to 100% (**Table 3**). Mixture 7 (equal proportion of the three EOs) exhibited the highest inhibition rate compared to other treatments, showing an average of 64.59% in C1 followed by C2 with 80.19%, whereas 100% inhibition was obtained at C3 compared to individual treatments which showed this value only at a higher concentration (C5).

#### 3.2.2 Simplex centroid design analysis

The matrix of simplex centroid design and results for IC_50_ are shown in **Table 4**. The IC_50_ was calculated based on mycelial growth inhibition. The results obtained showed that the lowest value (0.013 µL/mL) was obtained with mixture 7 containing the three EOs, while the highest value was obtained with C. citratus EOs (0.163 µL/mL).

**Table 4:** Matrix of simplex centroid design and results for IC_50_.

Mixture	*L. dentata*	*S. rosmarinus*	*C. citratus*	IC_50_ (µl/ml air)
1	1	0	0	0.107
2	0	1	0	0.116
3	0	0	1	0.163
4	0.5	0.5	0	0.066
5	0.5	0	0.5	0.055
6	0	0.5	0.5	0.054
7	0.33	0.33	0.33	0.013

The regression coefficients for the special model have been computed and are presented in **Table 5**. The associations between the tested parameters and the obtained responses for IC_50_ were found using regression models with significant coefficients (p-values < 0.05). Due to the inherent dependency among the components (X_1_ + X_2_ + X_3_ = 1), individual p-values for the linear coefficients (β_1_, β_2_, β_3_) were not estimable. Among the interaction terms, the coefficients of the ternary (β123) and binary (β12, β13, β23) models demonstrate statistical significance regarding the IC_50_ response. These interactions have a negative impact. In fact, the interactions between the factors reduce the value of the response variable IC_50_. The term L. dentata * S. rosmarinus * C. citratus has the highest negative effect among the interactions. However, the coefficients of individual components (β1, β2, β3) are non-significant (p > 0.05) and do not demonstrate an effect on the IC_50_.

**Table 5:** The regression coefficients of the model (nd; non-determined).

Term	Coefficients	Estimation	p-value
*L. dentata*	β1	0.107000	nd
*S. rosmarinus*	β2	0.116000	nd
*C. citratus*	β3	0.163000	nd
L. dentata*S. rosmarinus	β12	-0.18200	<0.001
L. dentata*C. citratus	β13	-0.32000	<0.001
S. rosmarinus*C. citratus	β23	-0.34200	<0.001

Equation 2 represents a mathematical model that describes the response as a function of the tested components.

(2) $$\begin{array}{c} \text{Y}=0.107X_{1}+0.116X_{2}+\;0.163X_{3}\;-0.182X_{1}X_{2}-\\ 0.32X_{1}X_{3}\;-0.342X_{2}X_{3}\;-0.591X_{1}X_{2}X_{3}\;+\;\epsilon \end{array}$$

The standard error is very small (S = 0.001), some p-values may not be calculated because there is not enough dispersion to detect significant differences (Case of individual mixtures). The Sum of Predicted Error Squares (0.0000315) is close to 0, indicating that all observations fit the model almost exactly. Moreover, R^2^ and R^2Adjusted^ show higher values (99.97 and 99.96, respectively) suggest strong agreement between the modeled and observed data. Additionally, the ANOVA F-tests confirmed the validity of the models, with p-value<0.001 and F = 7436 (**Table 6**).

**Table 6:** Model summary.

Model	DF	R^2^	R^2Adjusted^	MS	SS	F-value	p-value
Regression*	6	99.97%	99.96%	0.001	0.0000315	7436.00	<0.001

DF, degree of freedom; SS, sum of squares; MS, mean square; R², coefficient of determination. *Statistically significant at p < 0.05.

#### 3.2.3 Mixture profile

**Figure 2:**
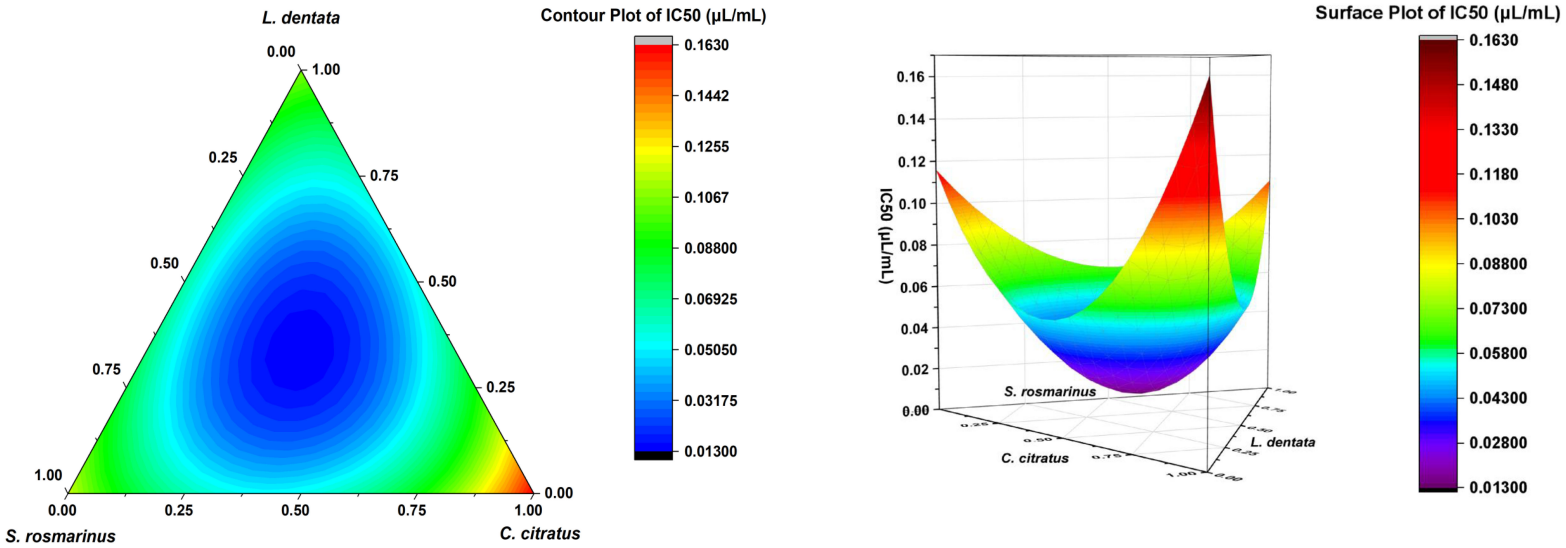
Results of IC_50_ and adjusted models.

The contour plot and 3D surface graph (illustrated as 2D and 3D mixture plots in **Figure 2**) demonstrate the optimal proportions of the three EOs mixture to maximize responses, thereby illustrating the relationship between responses and proportions of each EO. The creation of these plots was facilitated by Design-Expert software, which employs iso-response curves, thus ensuring optimal response values. The color scheme utilized in the plots offers a visual representation of the relationship between the responses and the proportion of each EO. Specifically, blue is indicative of lower IC_50_ values and higher antifungal activity, while the transition of colors from yellow to dark red signifies increasing IC_50_ values, thereby indicating reduced effectiveness.

### 3.3 Antifungal assay with the selected mixture

#### 3.3.1 *In vitro* direct contact assay

**Figure 3:**
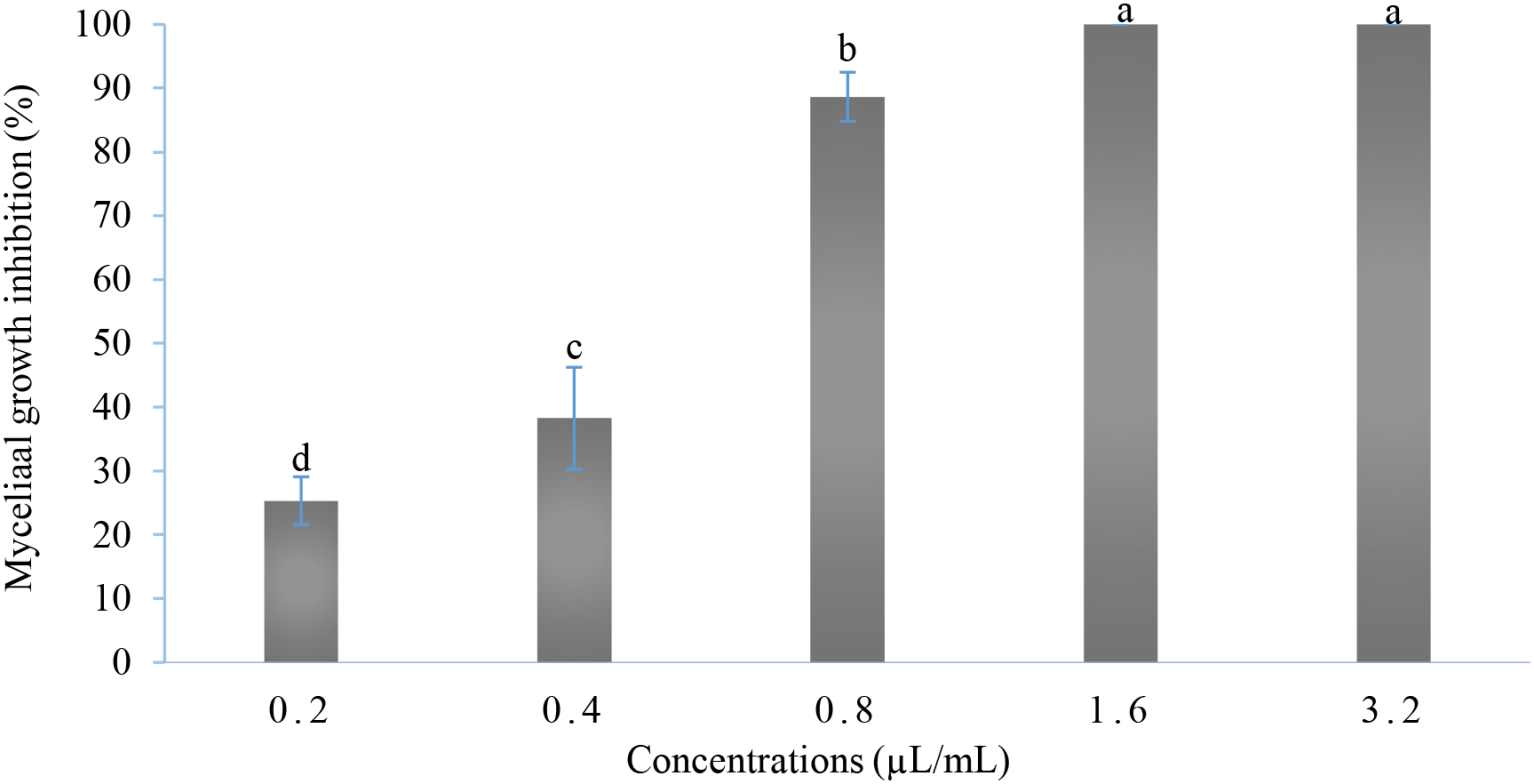
*In vitro* antifungal activity of the selected mixture. Values with the same letters are not significantly different according to the Kruskal-Wallis test followed by Dunn’s test at 5%.

Based on the results of simplex-centroid design analysis, the mixture 7 containing the three EOs was selected. As shown in **Figure 3**, the mycelial growth of *B. cinerea* was significantly reduced in all examined concentrations (P-value < 0.001). The highest inhibition rate was obtained with the concentrations 1.6 and 3.2 µL/mL reaching 100%, followed by the concentrations 0.8 and 0.4 µL/mL (88.65% and 38.28%, respectively). However, at the lowest concentration, the inhibition rate was 25.32%. Moreover, the IC_50_ and IC_90_ obtained were 0.46 and 0.81 µL/mL, respectively.

#### 3.3.2 MIC, MFC, and inhibition of spore germination

**Figure 4:**
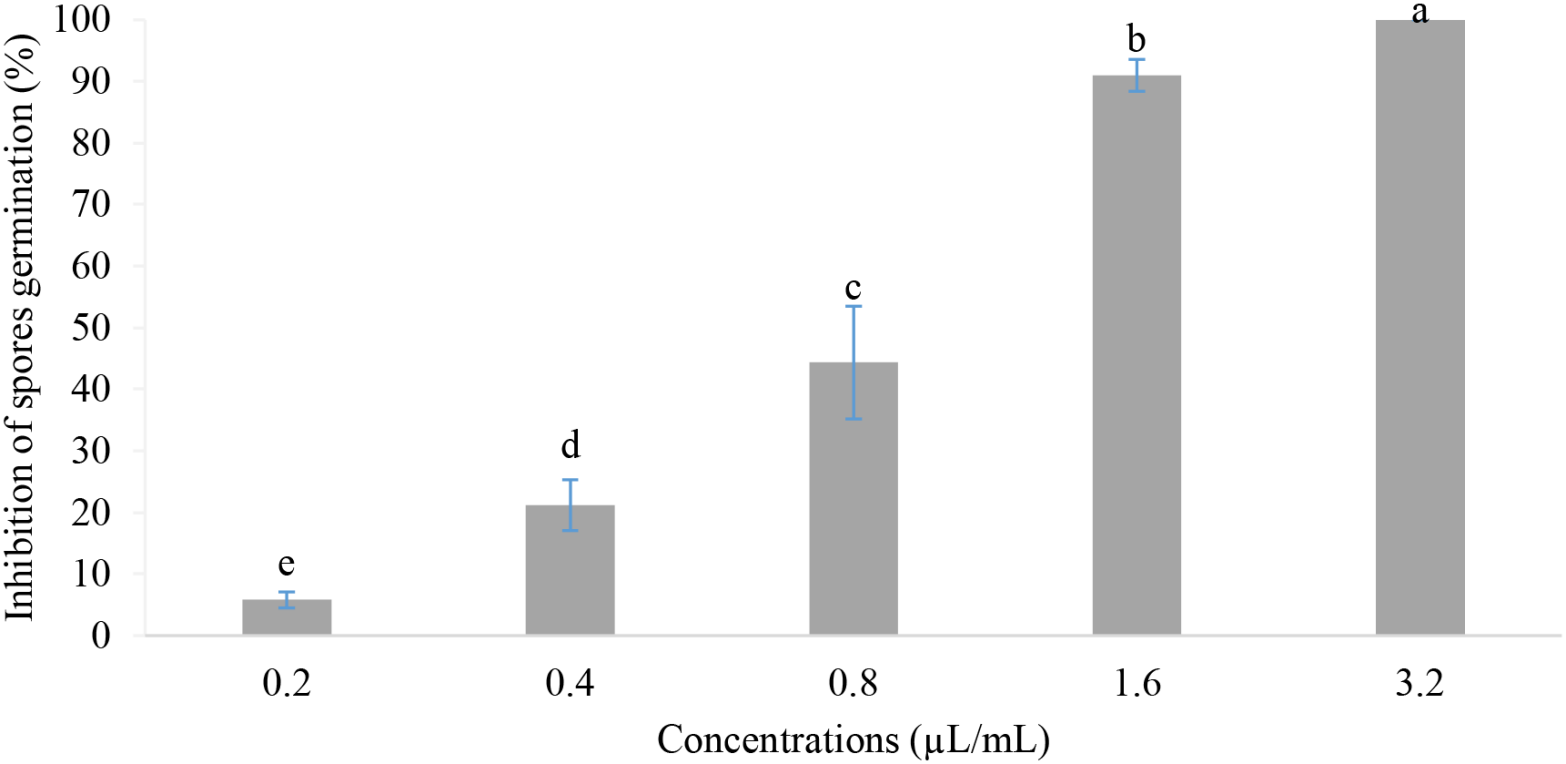
Spore germination inhibition *in vitro*. Values with the same letters are not significantly different according to the Kruskal-Wallis test followed by Dunn’s test at 5%.

Effects of different concentrations of the EOs mixture on spore germination of *B. cinerea* are shown in **Figure 4**. According to the results obtained, the concentrations tested showed a significant difference in the inhibition of spore germination (P-value<0.001). The inhibition rate proportionally increased with concentrations ranging from 5.79% to 100%. On the other hand, the minimum inhibitory concentration was found to be 1.6 µL/mL, while the minimum fungicidal concentration was higher than 3.2 µL/mL (MFC > 3.2 µL/mL).

#### 3.3.3 *In vivo* assay of selected mixture on cherry tomato fruits

**Table 7** summarizes the results of the antifungal activity *in vivo* of the selected mixture on cherry tomato inoculated with *B. cinerea*. IC_90_ of the mixture exhibited an antifungal activity *in vivo* with an average of 88.29%. Moreover, the fruit decay area for untreated fruits was 132.37 mm^2^, while for treated fruits, the fruit decay area was 25.33 mm^2^, revealing a range of reduction in fruit decay (P-value<0.001).

**Table 7:** Inhibition rate and fruit decay area of treatments on cherry tomato fruits.

Treatment	Inhibition rate	Fruit decay area*
Mixture	88.29 ± 8.62%	25.33 ± 3.83 mm^2 b^
Control	–	132.37 ± 13.65 mm^2 a^
P-value	–	P < 0.001

*Values with the same letters are not significantly different according to the Mann-Whitney U test at 5%.

#### 3.3.4 *In vivo* assay of selected mixture on tomato plants

**Figure 5:**
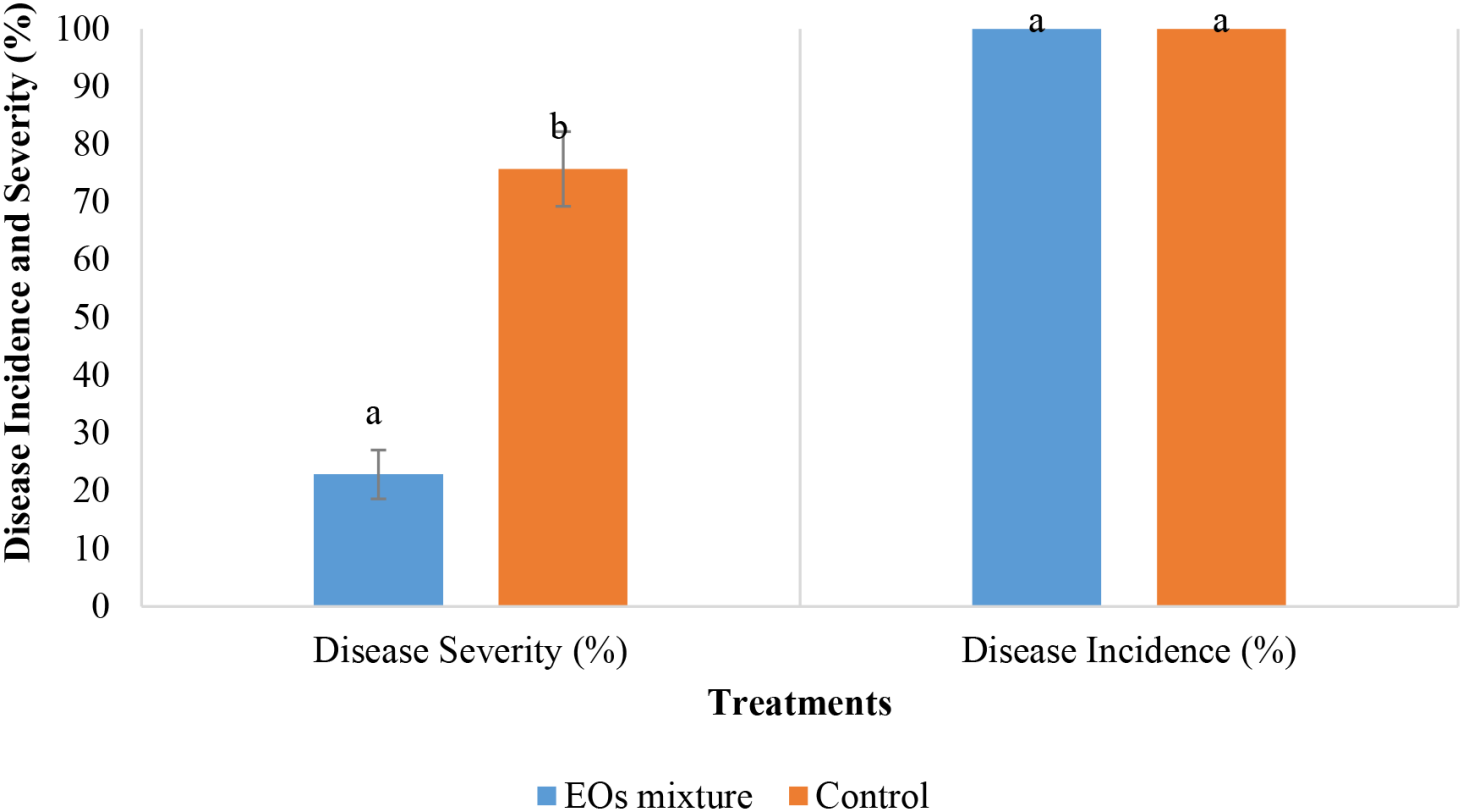
Effect of the mixture on the control of the grey mold caused by B. cinerea on tomato plants. Values with the same letters are not significantly different according to the Mann-Whitney U test at 5%.

**Figure 6:**
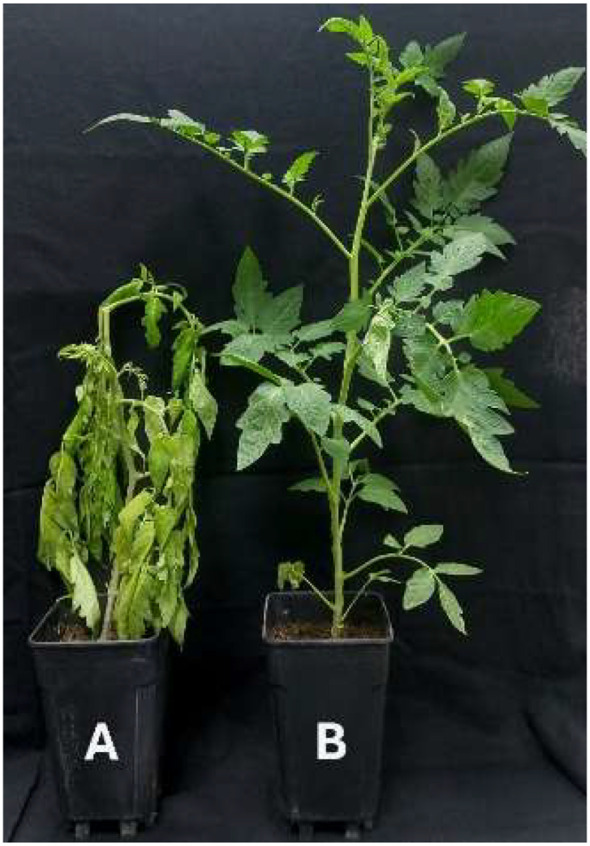
Effect of EOs on *B*. *cinerea* infested tomato plants. **(A)** Control and **(B)** Treated plant with EOs.

The results of the *in vivo* antifungal activity of the selected mixture on tomato plants inoculated with *B. cinerea* are demonstrated in **Figure 5**. After three weeks, all plants showed the development of *B. cinerea* symptoms with an average disease incidence of 100 ± 0.00%. However, the disease severity was significantly lower in plants treated with the EOs (22.86 ± 4.26%), compared with the control plants (P < 0.05), with a recorded value of 75.71 ± 6.46% (**Figure 6**).

## 4 Discussion

The present study explored the chemical profile and antifungal activity of EOs extracted from three medicinal and aromatic plants (*C. citratus, S. rosmarinus, and L. dentata*) against the phytopathogenic fungus *B. cinerea*. Through a combination of gas chromatography-mass spectrometry (GC-MS) profile analysis and *in vitro* antifungal assays, both individual and synergistic effects of the EOs were assessed. The results reveal not only the antifungal potential of these plant-derived oils but also demonstrate that their combination yields significantly improved inhibition, highlighting the value of synergistic interactions in botanical formulations.

GC-MS profiling of the tested EOs highlights both qualitative and quantitative differences in the major components, reflecting species-specific metabolic profiles. *C. citratus* EOs was predominantly composed of geranial, neral, and β-pinene. Our findings are consistent with the results obtained by other authors (He et al., 2023; Faria and Barbosa, 2024; Cherif et al., 2025; de Andrade Bomfim et al., 2025; Owolabi et al., 2025). Similar results were also found in a study previously conducted by Dangol et al. (2023), with a higher concentration of β-pinene compared to our results. *S. rosmarinus* was found rich in camphor, α-pinene, and 1,8-cineol; a comparable result to the previous study conducted by Cherif et al. (2025). However, the amount of 1.8-cineole was higher than the quantity found in our study (32.43%). Moreover, analogous results were described in other studies (Ait Bouzid et al., 2023; Annemer et al., 2023; Houzi et al., 2024; Ivanova et al., 2025; Xia et al., 2025). In contrast, Mozafari et al. (2024) found that the chemical composition of *S. rosmarinus* oils resulted in high concentration of 1,8-cineole, trans-caryophyllene, α and β-pinene; whereas Amiri et al. (2022) recorded camphene (33.8%) as the dominant compound in rosemary EO. Regarding *L. dentata* EOs, camphor, 1,8-cineol, and β-pinene were the most abundant compounds, in agreement with earlier reports (Cossetin et al., 2021; Ait Bouzid et al., 2023; Ait Elallem et al., 2024). The chemical analysis by Belcadi et al. (2024) revealed the same ranking, with a higher concentration of β-pinene. Different results were revealed by others. Akachoud et al. (2024) showed that camphor (28 %), terpinene-4-ol (12.14 %), and camphene hydrate (6.03 %) were the most abundant. Also, it was reported that 1,8-cineol, camphor, and fenchone were the major compounds of these EOs (Wagner et al., 2021; de Oliveira Xavier et al., 2024). This variability in the chemical profile of EOs may be attributed to several factors, including plant species, geographic origin, cultivation conditions, extraction method, and the developmental stage of the plant material (Mohamed Hanaa et al., 2012; Labhar et al., 2024; Jemai et al., 2025; Lahmar et al., 2025).

Furthermore, our findings revealed that the EOs of three plants exhibited varying levels of inhibitory activity of *B. cinerea* in a dose-dependent manner. This might be attributed to the mode of resistance behavior of the fungi against various substances present in EOs. For all plants, the concentration of 0.32 µL/mL air inhibited the mycelia growth of *B. cinerea* by 100%. These results corroborate those described by Aguilar-González et al. (2015), which showed that *Syzygium aromaticum* EO and *Brassica nigra* EO inhibited *B. cinerea* growth at a concentration of 92.56 μL/L air and 5.42 μL/L air, respectively. Amiri et al. (2022) found that *Cuminum cyminum* EO exhibited 100% inhibition in the vapor phase at 4 μL/mL, while EO of *S. rosmarinus*, *Citrus limon* and *Eucalyptus globulus* exhibited a complete inhibitory effect at 10 μL/mL, ranging between 41.1% and 40% inhibition rate for lemon and eucalyptus, respectively. In another study, the EOs of *Cymbopogon martinii* and *Mentha* sp*icata* showed the greatest efficacy against *B. cinerea*, with an inhibition rate of 100% in the volume of 5 µL, while *Cinnamomum camphora* and *Mentha×piperita* revealed the same activity at 10 µL (de Oliveira Filho et al., 2021). Previous investigations reported that secondary metabolites act against fungi through diverse mechanisms such as the disruption of the fungal cell membrane, the inhibition of cell wall synthesis, the disruption of cell division by inhibiting mitosis, and the inhibition of DNA and RNA synthesis. In this sense, Pedroso et al. (2024) assessed the antifungal activity and mechanism of action of monoterpenes against *B. cinerea.* The findings showed that carvacrol, citral, citronellal, citronellol, geraniol, and thymol demonstrated significant antifungal activity. Moreover, thymol and carvacrol induced conidial death, resulting in the disruption of cell membrane integrity, increased intracellular ROS levels, and decreased mitochondrial membrane potential. Singh et al. (2024) also showed that 1,8-cineole significantly reduced the ergosterol biosynthesis and downregulated genes related to chitin synthase, ergosterol synthase, cellulase production, and glycosyl hydrolase. Furthermore, Kong et al. (2022) revealed that camphor inhibited the growth of *Fusarium* and caused cytomembrane destruction, enhancing its permeability and releasing intracellular macromolecules, such as nucleic acids and proteins. Also, Zheng et al. (2021) reported that citral inhibits ergosterol biosynthesis and damages membranes in filamentous fungi. In addition, it’s reported that citral downregulated ochratoxin biosynthetic genes, and reduced the level of enzymes associated with respiration, resulting in the disruption of energy metabolism (Wang et al., 2021).

Although many studies report the antifungal activity of individual EOs, fewer have addressed the effects of EO mixtures, confirming that the overall activity of botanical extracts is a result of mixtures of compounds with synergistic, additive, or antagonistic activity (de Oliveira Filho et al., 2021; Gonçalves et al., 2022; Faria et al., 2023; Ben Akacha et al., 2024; Elbouzidi et al., 2024; Kaya et al., 2025; Owolabi et al., 2025; Zhu et al., 2025). Similarly, our study confirmed that the combination of EOs increased the antifungal activity of *B. cinerea*. All evaluated mixtures showed total inhibition of mycelial growth with the concentration C3 (0.08 µL/mL air of Petri dish) except for mixture 5 (*L. dentata* EOs + *C. citratus* EOs), which inhibited the mycelial growth at C4 (0.16 µL/mL air of Petri dish). This increase in antifungal activity is hypothesized to result from the complementary mechanisms of action of the EOs compounds. For instance, while phenolic compounds such as carvacrol and eugenol have been shown to destabilize membranes (Hyldgaard et al., 2015; Bai et al., 2022), aldehydes such as citral have been demonstrated to interfere with intracellular proteins and enzymatic systems, thus amplifying the overall antifungal effect (Cai et al., 2019). Our findings are strongly supported by prior studies investigating EO synergies. In a study, 1,8-cineole showed synergistic interactions with α-pinene, increasing the activity against the yellow fever mosquito *Aedes aegypti* (Sarma et al., 2019). Similar results were found by Aguilar-González et al. (2015) which confirmed the effect of the combination of clove EO with mustard EO in amplifying the inhibition of *B. cinerea* compared to their individual application. Furthermore, studies reported that the binary mixtures of *C. citratus* with *M. piperita* and of *Foeniculum vulgare* with *Satureja montana* EOs against the Pinewood Nematode *Bursaphelenchus xylophilus* resulted in higher activity compared to the individual treatments (Gonçalves et al., 2022; Faria et al., 2023). Nevertheless, the combination of certain bioactive compounds may possess antagonistic interactions in comparison to each compound separately. Sarma et al. (2019) reported that 1,8-cineole showed mainly antagonistic interactions with other monoterpenes, e.g., carvone and limonene, while no interaction was noticed between α-pinene and other terpenes. In another study, Hossain et al. (2016) reported that the use of EOs of *Citrus reticulate* and *Eucalyptus globulus* combined with EOs of *Thymus vulgaris* and *Origanum vulgare*, respectively, generated no improvement in antifungal activity against *Aspergillus niger*, *A. flavus*, *A. parasiticus* and *Penicillium chrysogenum*. The same was observed by de Oliveira Filho et al. (2021) where the combination of EOs of *M. piperita*, *C. martinii*, *C. camphora*, and *M.* sp*icata* as binary mixtures maintains a similar level of antifungal activity against *B. cinerea* compared to pure EOs.

Based on the simplex-centroid design analysis and the ranking of mixtures regarding their *in vitro* antifungal activity by the method of exposure to volatiles, the mixture 7 which contains *L. dentata*, *S. rosmarinus*, and *C. citratus* EOs at a ratio (1:1:1) was selected. After exposure by contact at different concentrations of this mixture, the mycelial growth was completely inhibited at 1.6 µL/mL and 3.2 µL/mL, while the IC_50_ and IC_90_ obtained were 0.46 µL/mL and 0.81 µL/mL, respectively. Moreover, the values of MIC and MFC were respectively 1.6 µL/mL and >3.2 µL/mL. Compared to other studies, de Oliveira Filho et al. (2021) showed that binary mixtures of *M. piperita* - C*. martini* and *C. camphora* - *M.* sp*icata* inhibited the mycelial growth of *B. cinerea* at the concentrations of 500 and 750 µL/L. Many studies demonstrated the inhibition capability of EOs of postharvest fungi under *in vitro* conditions; however, few studies have been conducted on the *in vivo* efficacy of EOs. In this study, the optimal mixture of the three EOs showed a strong *in vivo* antifungal activity against *B. cinerea*. The decay of cherry tomatoes was inhibited with an average of 88.29%. Also, the disease severity value recorded for the plant treated with the EOs mixture was 22.86% compared to the control, with an average of 75.71%. Similarly, Brito et al. (2021) confirmed that the use of mixtures of EOs of *Origanum vulgare*, *Thymus vulgaris*, *Citrus limon*, and *Citrus sinensis* and their respective hydrolats enhanced the antifungal activity on *B. cinerea* inoculum on tomato fruits. In another study, Zhao et al. (2021) found a 70% reduction in the decay of cherry tomatoes with the use of *O. vulgare* EOs at 62.5 mg/L, compared to untreated samples, while at 250 mg/L, the reduction in the decay of fruits reached 96%. On the other hand, He et al. (2023) demonstrated the efficiency of *C. citrates* EOs at 125 mg/L, indicating the reduction of the decay area of cherry tomatoes by more than 90%. Regarding *in vivo* tests on plants, Soylu et al. (2010) evaluated the curative and protective effects of different concentrations of *Origanum syriacum* L. var. bevanii EO on the infection caused by *B. cinerea* in the greenhouse. The finding showed that the disease index at 75 mg/L of *O. syriacum* L. var. bevanii EOs was 77% and 33% in curative and protective effects, respectively.

To reiterate, the combination of EOs increased the *in vitro* antifungal activity compared to their individual application. Furthermore, the mixture containing the tested EOs has been shown to control infection of tomato fruits and plants by *B. cinerea* under greenhouse conditions. This interaction among the EOs not only enhances antifungal efficacy but also allows for reduced concentrations, offering both biological and economic advantages. These findings support the development of novel, plant-based fungicidal formulations and contribute to the growing body of research advocating for natural solutions in plant disease management.

## 5 Conclusion

The current study demonstrates the promising antifungal potential of EOs extracted from *C. citratus*, *S. rosmarinus*, and *L. dentata*, both alone and in combination, against B. cinerea, a major tomato pathogen. The positive effects observed when combining EO mixtures significantly increased antifungal efficacy, lowering the required effective doses and demonstrating their practical advantage over individual applications. The findings highlight the potential of these EO mixtures as a viable alternative to traditional fungicides. Furthermore, their efficacy at low concentrations may help to reduce the selective pressure for pathogen resistance development, thereby improving the long-term viability of crop protection strategies. The use of eco-friendly EO mixtures can have a significant impact on agroecological operations. These combinations are effective at increasing biodiversity, lowering chemical inputs, and addressing the environmental and health risks associated with synthetic pesticides. The ability of mixtures to function through numerous routes makes them an important tool in integrated disease management. Future studies should focus on formulations and their stability, as well as the cost sustainability of EO-based treatments in field settings. This ensures widespread use within agroecological and organic farming systems.

## Data Availability

The original contributions presented in the study are included in the article/supplementary material. Further inquiries can be directed to the corresponding author.
